# Detection of neuroglobin in umbilical cord blood signals progress in perinatal medicine

**DOI:** 10.1038/s41598-025-11733-0

**Published:** 2025-07-25

**Authors:** L. Filonzi, A. Ardenghi, D. Ponzi, A. Bolchi, Y. Ciummo, S. Paterlini, V. Beretta, V. Dell’Orto, M. T. Bruno, P. Palanza, F. Nonnis Marzano, S. Perrone

**Affiliations:** 1 Department of Chemistry, Life Sciences and Environmental Sustainability, University of Parma, Viale delle Scienze 11, Parma, 43124 Italy; 2 Department of Medicine and Surgery, University of Parma, Via Gramsci 14, Parma, 43126 Italy; 3 Neonatology Unit, University Hospital of Parma, Via Gramsci 14, Parma, 43126 Italy

**Keywords:** Hypoxia, Molecular markers, Neonatology, Neuroprotection, Oxidative stress, Molecular neuroscience, Predictive markers

## Abstract

To assess whether Neuroglobin could play a functional role during perinatal period and birth, it was analyzed in 83 umbilical cord blood samples where its concentration ranged between 1.65 and 45.18 ng/mL, mean 18.49 ng/mL. Although resembling concentrations previously detected in many pathologic conditions in adults, none of newborns displayed altered Apgar score and were regularly discharged in healthy status. Surprisingly, 83.13% of babies had NGB concentrations higher than the putative 8.4 ng/mL value, recently hypothesized as a prognostic cut-off between good and bad recovery from cerebral ischemia in adults. Significant Pearson correlations were observed between NGB and Hb (*r* = 0.368, *p* = 0.001), and Htc (*r* = 0.372, *p* = 0.001) confirming its physiological role in oxygen-regulated metabolic information within the child-mother dyad. Besides the direct action in regulating blood flow and gas exchange, the first NGB discovery in cord blood is discussed in relation to new perspectives in perinatal medicine.

## Introduction

An increasing interest has been dedicated to Neuroglobin (NGB) since its first discovery as neuronal globin in the pyramidal cells of mammals in 2000^1,2^. Since then, major attention has been dedicated to its structure, expression pattern and localization to clarify structure-function relationships in a wide variety of vertebrates, from fish to humans^3–6^.

In vivo experiments in different species have highlighted its crucial role in maintaining oxygen homeostasis in the brain. In fact, neuroglobin overexpression significantly protects both heart and brain from hypoxic/ischemic and oxidative stress-related insults, whereas decreased NGB levels may lead to an exacerbation of tissue injuries^7^. Its importance for the preservation of vitally fundamental functions is confirmed by its early evolutionary origin and conserved characteristics in many taxa throughout the nervous system evolution^5,8^.

A positive correlation between ischemia and NGB expression levels was confirmed in humans. Analyses of postmortem brain tissues derived from patients who suffered from ischemic stroke revealed the increase of the NGB expression in the cortex near the infarct area with respect to the adjacent normal brain and the ischemic center^9^.

On the other hand, the serum level of NGB significantly increases after pathological changes in the brain, as reported in the gerbil brain after ischemic insults^10^, in Sprague Dawley rats with sepsis-associated encephalopathy^11^, and in human subjects with obstructive sleep apnea^12^ or after traumatic brain injury^13^. The impairment of the blood–brain barrier as well as the damage of neuronal membrane integrity may result in the release of the NGB protein into the blood, rendering serum NGB levels as a possible biomarker for predicting severity and prognosis of brain pathologies^13^.

From a functional point of view, different single nucleotide polymorphisms (SNPs) and biomolecules regulate its gene expression. In particular, the mechanism responsible for hypoxia-induced NGB upregulation involves the transcription factor HIF-1α^14,15^. An important role at epigenetic level is also played by 17 β-Estradiol (E2)^16^. Consequently, the first intronic enhancer may shift from poised to the active state in neuronal cells after E2 in vitro stimulation^16^. This aspect should be further investigated during pregnancy.

Although the highest mRNA expression was observed in the central and peripheral nervous system^7^, comparatively less expression has been detected in other non-neuronal tissues where the protein functional role is still unclear^17^. Hypotheses were formulated on its concentration-dependent functional role. More precisely, when present at relatively low concentrations (around 1 µM), including resting neurons, its contribution to oxygen supply and/or facilitated diffusion is insignificant^18^. On the opposite, human neuroglobin is present at relatively high concentrations in metabolically active cells and certain specialized ones, such as hypothalamic neurons and retinal cells. In these cells, its concentration has been estimated to reach up to 100 µM^17,19^.

However, it is noteworthy observing that while NGB appears to mainly participate in the cellular defense against hypoxia, the different responses between in-vivo and in-vitro experiments are contradictory^20^. In fact, several studies of oxygen deprivation in vivo suggested that NGB synthesis is not stimulated after mild hypoxia^21^. On the other hand, as a mitochondrial (or alternatively as peri-mitochondrial) scavenger of ROS and RNS, NGB could play a fundamental role in protecting against ischemic injuries. In fact, several studies suggested that an interaction between NGB and cytochrome c is a protective mechanism able to prevent apoptosis under oxidative and nitrosative stress through cyt c reduction^22–24^. This aspect would highlight the citoprotection role of NGB, rather than being a mere O_2_ sensor. Globally, it must be remarked that the different responses obtained in cultured cells and in vivo remain to be fully clarified^20^.

Reflecting on the above cited aspects, more than 20 years of scientific research on NGB has generated an amount of data and knowledge about this promising globin. Despite this, several aspects of its biochemistry and molecular functions still remain unclear and must be further debated. To the best of our knowledge, no publications are so far available on the possible neuroprotective role of NGB during human fetal life. Interestingly, a possible correlation between brain maturation stage, severity of hypoxia, NGB levels, and neuron survival was observed in the developing mouse and rat brains^15,25^.

Considering that no reliable data nowadays exist regarding NGB concentration in the human fetus and newborns, an investigation was carried out to unravel whether NGB could play a functional role during the perinatal period and delivery. More precisely, the aim of this study was to examine the concentration of NGB in the umbilical cord blood of healthy term newborn infants for the first time, and to associate results to the assessed clinical parameters evaluated at delivery. This work opens important perspectives on the role of Neuroglobin in the perinatal life stage, with a special focus on its possible buffering role during adaptive states triggered by pathological hypoxic transitions, both as an oxygen sensor and as regulator of arterial perfusion.

## Materials and methods

Eighty-three newborn babies with gestational age ranging between 37 and 41 weeks, consecutively admitted to the Neonatology Unit of Parma, University Hospital, were enrolled in the study. Clinical characteristics of enrolled population are reported in Table 1. The study was approved by the Local Ethical committee, protocol code 42/2020/SPER/UNIPR, and informed written consent was obtained from parents of all enrolled babies. All experiments were performed in accordance with relevant guidelines and regulations issued by the Medical School of Parma University.

Newborns with congenital malformations (*n* = 3), inborn errors of metabolism (*n* = 1), feto-maternal blood group incompatibility (*n* = 4), sepsis (*n* = 1), multiple gestation (*n* = 2), and those with clinical signs of perinatal hypoxia (*n* = 6), were excluded. In particular, perinatal hypoxia was defined as the presence of at least two of the following conditions: intrapartum distress, as indicated by fetal bradycardia with a heart rate of less than 100 beats per minute; an Apgar score of 6 or less at five minutes; a need for resuscitation for more than one minute with positive-pressure ventilation and oxygen supplied immediately after birth; a pH value of 7.10 or less in umbilical artery. Five parents did not release the informed consent and were therefore similarly excluded from the final dataset.

Blood samples (2 mL volume) were obtained from the umbilical artery, within 60 s after cord clamping. Insufficient liquid tissue was collected in 3 cases. Blood was centrifuged within 1 h from sampling to avoid the effects of storage. After centrifuging at 1,000xg at 2–8 °C, both plasma and serum were collected to make appropriate analytical comparisons for NGB concentration. Plasma and serum samples were then stored in a −80 °C until analysis. It is noteworthy observing that although not statistically evaluated, no analytical difference emerged between plasma and serum during comparisons. Plasma was therefore preferred to assess NGB concentration (referred both as ng/mL and µM) since serum was addressed to a different topic of research.

To detect the NGB content in each sample, a sandwich ELISA test was conducted using Neuroglobin (NGB) ELISA kit (BIOMATIK, cat #EKU06219), in accordance with the recommendations of the producer (BIOMATIK USA Wilmington, Delaware). A subsample of 10 uL obtained from each thawed plasma sample was initially diluted at 1:10 and subsequently brought to a final dilution of 1:200. One hundred microliters of each 1:200 diluted sample were hence submitted to ELISA analysis.

The concentration of NGB was determined by comparing OD value of the sample, measured at 450 nm, to the standard curve, that was constructed using the standard NGB provided by the kit. Specifically, the concentration of NGB in ng/mL was calculated for each sample from OD value, subtracted from the blank value, using the equation of the standard curve. The concentration value was multiplied by the dilution factor. Analyses were carried out in triplicate and final concentration was obtained by calculating the mean value for each sample (after a preliminary survey to assess consistency among replicate measures).

Besides NGB, the Apgar score and chemical-clinical data were also obtained. In particular, biochemical analyses focused on 8 additional functional parameters (Table 2). Pearson’s correlations were used to explore the possible association between NGB and the 8 functional parameters. Considering that a limited number of parameters were randomly omitted during registration in the clinical records, we report the sample size for each measurement. Data are presented as mean ± standard deviation.

## Results

The population study consisted of 83 mothers and their healthy newborns, born at term of gestational age. Sixty of the 83 mothers (72.23%) were primiparas and 78 (93.97%) were of Caucasian ethnicity. Mean gestational age was 39.65 weeks and newborns birthweight was in the normal range (M = 3.38 Kg; SD = 0.40). Apgar scores were in the range 8–10 at 10 min, mean value 9.88.

NGB plasma levels ranged between 1.65 and 45.18 ng/mL, mean concentration 18.49 ng/mL (SD = 10.37). No difference emerged between males (M = 18.26; SD = 10.32) and females (M = 17.82; SD = 9.34) regards to arterial cord blood NGB concentrations.

**Table 1:** Descriptive anamnestic and clinical data of newborns.

Outcome (*n* = 83)	Mean ± SD
Gestational age (weeks)	39.65 ± 1.18
Body weight (g)	3,385 ± 403.50
Body length (cm)	51 ± 2.15
Cranial circumference (cm)	35 ± 1.34
**Delivery**	**Number of cases (%)**
Spontaneous	64 (77.11)
Elective cesarean section	8 (9.63)
Emergency cesarean section	6 (7.23)
Vacuum delivery	5 (6.03)
Primiparous mother	60 (72.28)
**Fetal presentation**	
Vertex	78 (93.98)
Shoulder	1 (1.20)
Breech	3 (3.62)
Brow	1 (1.20)
Amniotic liquid (clear)	68 (81.92)

**Fig. 1:**
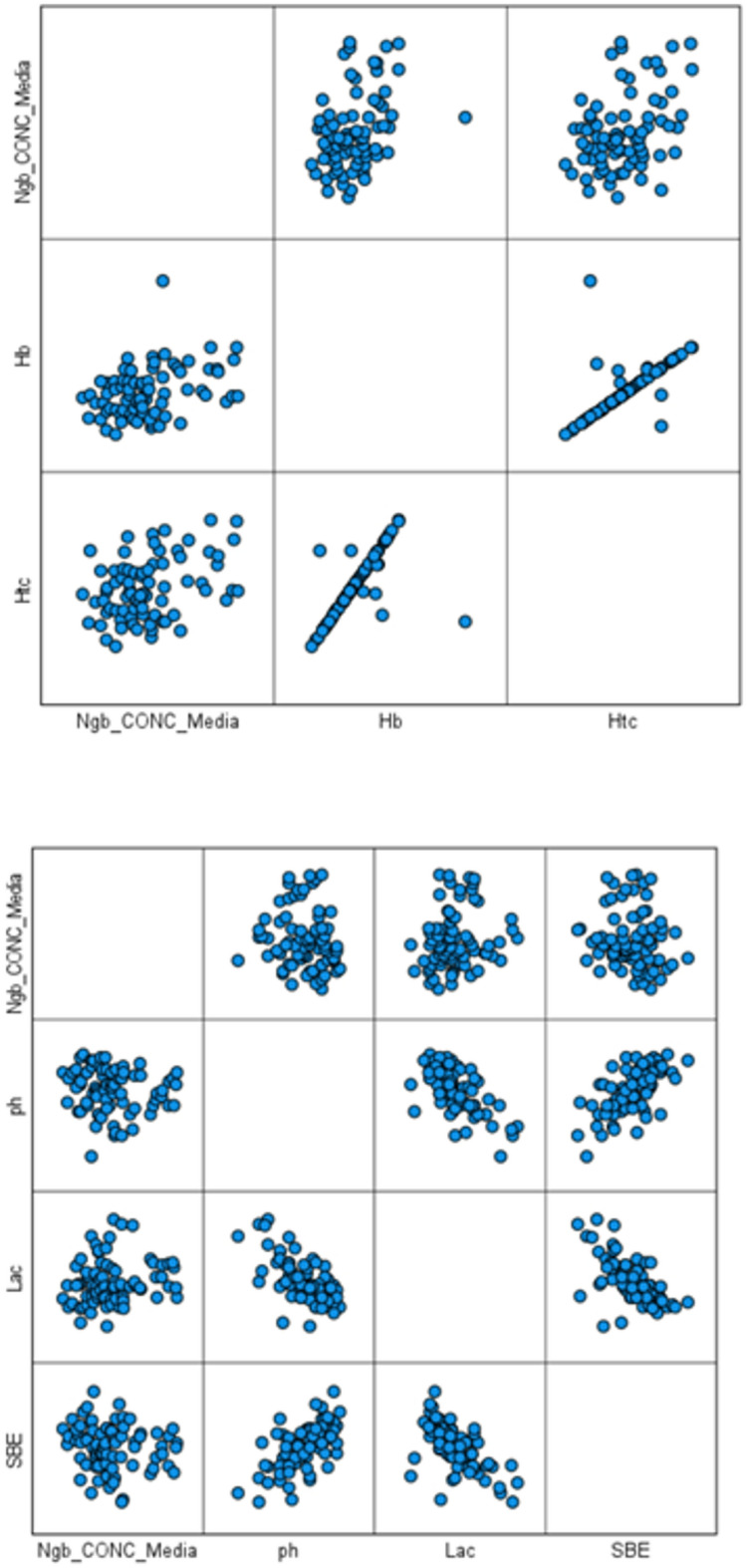
Graphical representation of Pearson correlations determined among biochemical parameters.

In addition to plasma NGB concentration, an analysis was also performed on other functional blood parameters, such as pH, arterial carbon dioxide tension (pCO₂), arterial oxygen tension (paO₂), hemoglobin (Hb), hematocrit (Hct), bilirubin (Bil), lactate (Lac), and base excess (SBE). Table 2 shows the biochemical measurements for the different considered parameters. The Pearson correlation among these eight physiological variables and neuroglobin (NGB) concentrations (Table 3) revealed statistically significant positive correlations between NGB concentrations and hemoglobin (*n* = 75, *r* = 0.368, *p* = 0.001) and between NGB and hematocrit (*n* = 75, *r* = 0.372, *p* = 0.001) in cord blood. Graphical representations of Pearson correlations among all parameters considered are illustrated in Fig. 1.

**Table 2:** Summary of biochemical measurements determined in a variable number (n) of patients in relation to availability of clinical records. NGB was assessed in all 83 babies.

	*n*	Mean (SD)	Min-Max
pH	79	7.25 (0.07)	7.03–7.37
pCO_2_ (mmHg)	78	51.07 (10.13)	32.10–83.30
paO_2_ (mmHg)	77	25.59 (8.41)	10.40–50.60
Hb (g/dL)	75	17.32 (1.84)	11.30–24.20
Htc (%)	75	52.52 (4.68)	43.60–63.00
Bil (mg/dL)	75	1.82 (0.61)	0.50–3.30
Lac (mg/dL)	76	4.58 (1.85)	0.90–9.80
SBE (mmol/L)	78	−4.80 (3.70)	−13.90-4.50
NGB (ng/mL)	83	18.49 (10.37)	1.65–45.18

pCO_2_: arterial Carbon dioxide tension; paO_2_: arterial oxygen tension; Hb: Hemoglobin; Htc: Hematocrit; Bil: Bilirubin; Lac: Lactate; SBE: Base excess; NGB: Neuroglobin.

**Table 3:** Correlations among 8 physiological variables and neuroglobin based on pearson correlation coefficient.

Variable	1	2	3	4	5	6	7	8	9
1. pH	-								
2. pCO_2_	−0.71**	-							
3. paO_2_	−0.03	−0.21	-						
4. Hb	−0.17	0.19	−0.05	-					
5. Htc	−0.15	0.20	−0.03	0.68**	-				
6. Bil	0.09	−0.05	0.01	0.12	0.27*	-			
7. Lac	−0.66**	0.32**	0.15	0.12	0.08	−0.09	-		
8. SBE	0.64**	−0.01	−0.24*	−0.01	0.02	0.12	−0.61**	-	
9. NGB	−0.12	0.03	−0.07	0.36**	0.37**	−0.08	0.18	−0.14	-

* 0 < 0.05; **p < 0.01.

## Discussion

Neuroglobin was the third vertebrate globin to be discovered and so named because its expression was first observed in the brain of humans and mice^1^. Its role in controlling blood perfusion, redox regulation, O_2_ levels, and NO signaling in vascular coupling to the nervous system has been hypothesized starting from its relatively high expression in the central and peripheral nervous systems^20^. However, the prominent protein role in the different tissues is still debated and further knowledge is necessary to solve the current dispute.

To positively contribute to the discussion, in this work NGB expression was investigated at birth, starting from the consideration that the perinatal period is somewhat a subclinical state under strict control of several buffering systems. It is noteworthy observing that a physiologic oxidative stress is essential during pregnancy to promote appropriate growth and development. In normal pregnancies, the earliest stages of development take place under hypoxic conditions of the early gestational sac to protect the developing fetus against the deleterious and teratogenic effects of O2 free radicals^26^. Hypoxic environment of the first trimester is also believed to play an important role in the regulation of trophoblast differentiation^27^. On the other hand, the neuroglobin upregulation induced by 17 β-Estradiol seems to prevent H_2_O_2_-induced apoptosis through cytochrome c mitochondrial control^16,22^.

According to Ascenzi et al.^7^, few experiments have addressed the role of NGB in development and ageing, while no data is so far available on such state as human pregnancy. Despite this, a limited number of preclinical studies suggest the importance of NGB under experimental conditions in rats pregnancy^25,28^ and human embryogenesis^29^. In one specific work, NGB was measured in human embryonic stem cells during neuronal differentiation in vitro, where it resulted barely detectable^29^.

In this research, NGB was found for the first time in the umbilical cord blood of all investigated infants born at term of gestational age. No previous data has ever been reported on NGB detection at the interface between term intrauterine stage and birth delivery. Considering the fetal pertinence of the arterial umbilical cord, the relevance of NGB in its flowing blood represents an important finding that stimulates additional evaluations and opens new perspectives in perinatal medicine. In fact, the fetal tissue of the umbilical cord serves not only as a vital conduit for fetal nourishment but also as a biological archive of intrauterine conditions, potentially offering valuable insights into fetal well-being to be assessed by neonatologists.

Although our experimental concentrations fell within ranges of previous publications^7,17,20^, the highest detected levels exceeded the value of 30 ng/mL measured by Chen et al.^13^ 48 h after traumatic brain injury in patients with compromised blood-brain barrier, or alternatively 11 ng/mL in cases with subarachnoid hemorrhage^30^. Interestingly, in most babies our concentrations resembled those of pathologic conditions but none of the infants displayed altered Apgar score (neither at 1, 5 nor at 10 min) and were regularly discharged from the hospital. Surprisingly, 83.13% of our babies had NGB concentrations higher than the value of 8.4 ng/mL recently assumed by Ding et al.^31^ as a prognostic cut-off between good and bad recovery from cerebral ischemia in adults. We are aware that it is not appropriate to compare values in adults with those in cord blood, also considering that brain injury in adults has a different biochemistry than in fetuses where the immature blood-brain barrier allows substances to pass more easily. Despite this, a general comparison may be worthful due to the lack of scientific data on this topic.

It must be remarked from previous literature in vertebrates that when present at relatively low concentrations (around 1 µM), NGB contribution to oxygen supply and/or facilitated diffusion is insignificant^18^. On the opposite, human neuroglobin is present at relatively high concentrations in metabolically active and specialized cells (neurons of the central and peripheral nervous system), where its value can easily reach 100–200 µM in the retinal ganglion cell layer^17,19^. The protein has also been determined in cultured cells of human glioblastoma and astrocytes, where concentrations tended to increase up to 100 times under variable experimental conditions. From this point of view, human NGB displays a protective role by scavenging NO in the presence of high O_2_ levels. Nonetheless, at low O_2_ concentrations, human NGB acts as a nitrite reductase producing NO, thus regulating intra-cellular hypoxic NO signaling pathways. Its expression is also moderately induced by hypoxia, H_2_O_2_ toxicity, vascular endothelial growth factor and polysaccharide^19^. These data suggest that human NGB is a stress-inducible protein that behaves as a compensatory protein responding to injuring cellular stimuli. Interestingly, NGB cope with cerebral hypoxia in diving mammals by either facilitating oxygen supply or protecting from reactive oxygen species. Therefore, results obtained on the NGB neuroprotective function are promising and studies on interacting additional human proteins may be of great value for defining the mechanisms underlying NGB activities^17^.

In this experimental work, the observed significant correlations between NGB and hemoglobin (Hb) and hematocrit (Htc) possibly suggest a role of this protein in the physiology of the fetal circulation, and in the oxygen-regulated metabolic information about neonates and their interactions with the mothers. In particular, Hb and Htc fluctuations positively correlated to NGB variability seem to be able to maintain a homeostatic control of pCO_2_ and paO_2_ in the fetus-mother connection. In fact, fetal health status depends on the respiratory and metabolic ability of placenta, as the crucial interface between mother and fetus. More precisely, the fetal circulation is an entirely transient event, not replicated at any point in later life, and functionally distinct from the pediatric and adult circulations. The central unique feature of the fetal circulation is that gas exchange does not occur in the lungs, but via the placenta. Oxygenated blood returns to the fetus from the placenta via a single umbilical vein^32^. The umbilical vein reflects maternal status and placental function, while the umbilical artery reflects fetal oxygenation and acid - base status^33^. Moreover, arteriovenous oxygen differences reflect placental respiratory function independently from gestational age, birth weight, duration of labor, and mode of delivery^34^.

In our study, the elevated NGB concentrations (according to the compared above cited data in pathologic adults) suggest a primary direct role of the protein to allow both a functional blood flow and gas exchange in normal pregnancy. On the other hand, a second fundamental aspect could be related to neuronal cell death and consequent NGB release. In fact, according to Castillo-Ruiz et al.^35^ half of the neurons initially generated in the mammalian brain are eliminated and this process occurs perinatally in many species. For the authors, birth may play a role in shaping cell death. Although the importance of this process has been recognized for over 50 years, many basic questions still remain, particularly in humans where experimental perinatology is difficult^35,36^. As a matter of fact, studies in adults demonstrated that the damage of neuronal membrane integrity may result in the release of the NGB protein into the blood, rendering serum NGB levels as a possible biomarker for predicting severity and prognosis of brain pathologies^13,20^. Interestingly, NGB also promotes neurogenesis through Wnt signaling pathway^37^. In mammalian evolution, humans represent an exception being characterized by a series of morphological changes for erect posture and bipedal locomotion. The remodeling and reduction of the pelvis together with the rapid and steep increase of brain volume in *Homo sapiens* evolution has generated a conflict of functions resulting in a great health risk for mothers and newborns during parturition. It is possible that perinatal NGB release could have evolved as an adaptation to protect the developing brain from anoxia. Comparative studies on cord blood NGB in different Primate species, or also in other mammals, should be carried out to assess this hypothesis and definitely assess the neuroprotective role of NGB under stressing conditions.

The limitation of our paper certainly relies in the relatively low number of cases; however, this is the first study demonstrating plasma concentrations of neuroglobin in cord blood. The detection of this protein appears of paramount importance for a better understanding of the physiological processes at the feto-maternal interface, particularly during biochemical modifications driven by hypoxic states. To date, no clinical studies have investigated whether this protein is altered during the perinatal period, a time when hypoxic-ischemic events may occur and potentially lead to adverse outcomes in newborns. As a matter of fact, the possible role of NGB as a neuro-biomarker in neonates suffering serious brain injury issues is herein hypothesized and will have to be deeply investigated in the future to pursue in-depth knowledge for prevention or better cures to be applied during early life stages, particularly in light of emerging insights into the therapeutic potential of this globin^38,39^.

## Data Availability

The datasets used and/or analysed during the current study available from the corresponding author on reasonable request.
